# Preoperative and Noninvasive Prediction of Gliomas Histopathological Grades and IDH Molecular Types Using Multiple MRI Characteristics

**DOI:** 10.3389/fonc.2022.873839

**Published:** 2022-05-27

**Authors:** Ningfang Du, Xiaotao Zhou, Renling Mao, Weiquan Shu, Li Xiao, Yao Ye, Xinxin Xu, Yilang Shen, Guangwu Lin, Xuhao Fang, Shihong Li

**Affiliations:** ^1^ Department of Radiology, Huadong Hospital, Fudan University, Shanghai, China; ^2^ Department of Emergency, Changhai Hospital, Naval Medical University, Second Military Medical University, Shanghai, China; ^3^ Department of Neurosurgery, Huadong Hospital, Fudan University, Shanghai, China; ^4^ Department of Pathology, Huadong Hospital, Fudan University, Shanghai, China; ^5^ Clinical Research Center for Gerontology, Huadong Hospital, Fudan University, Shanghai, China; ^6^ Institute of Business Analytics, Adelphi University, Garden City, NY, United States

**Keywords:** glioma, magnetic resonance imaging, isocitrate dehydrogenase, diffusion-weighted magnetic resonance imaging, apparent diffusion coefficient

## Abstract

**Background and Purpose:**

Gliomas are one of the most common tumors in the central nervous system. This study aimed to explore the correlation between MRI morphological characteristics, apparent diffusion coefficient (ADC) parameters and pathological grades, as well as IDH gene phenotypes of gliomas.

**Methods:**

Preoperative MRI data from 166 glioma patients with pathological confirmation were retrospectively analyzed to compare the differences of MRI characteristics and ADC parameters between the low-grade and high-grade gliomas (LGGs vs. HGGs), IDH mutant and wild-type gliomas (IDH^mut^ vs. IDH^wt^). Multivariate models were constructed to predict the pathological grades and IDH gene phenotypes of gliomas and the performance was assessed by the receiver operating characteristic (ROC) analysis.

**Results:**

Two multivariable logistic regression models were developed by incorporating age, ADC parameters, and MRI morphological characteristics to predict pathological grades, and IDH gene phenotypes of gliomas, respectively. The Noninvasive Grading Model classified tumor grades with areas under the ROC curve (AUROC) of 0.934 (95% CI=0.895-0.973), sensitivity of 91.2%, and specificity of 78.6%. The Noninvasive IDH Genotyping Model differentiated IDH types with an AUROC of 0.857 (95% CI=0.787-0.926), sensitivity of 88.2%, and specificity of 63.8%.

**Conclusion:**

MRI features were correlated with glioma grades and IDH mutation status. Multivariable logistic regression models combined with MRI morphological characteristics and ADC parameters may provide a noninvasive and preoperative approach to predict glioma grades and IDH mutation status.

## Introduction

Glioma is the most common primary tumor in the central nervous system. Clinically, glioma is usually divided into low-grade gliomas (LGGs) and high-grade gliomas (HGGs) based on the histopathological assessment. LGGs are well-differentiated, while HGGs are poorly differentiated and have a relatively poor prognosis (1–3). In recent years, more and more studies have shown that traditional histopathological grading of glioma has certain limitations due to the remarkable heterogeneity of tumors. For example, some LGGs overlap genetically with primary glioblastoma and show similar rapid disease progression (4, 5). It is difficult to distinguish them just by evaluating proliferation markers and cell morphology (6).

The 2016 World Health Organization (WHO) Classification of Tumors of the Central Nervous System officially listed molecular detection results as one of the important diagnostic bases for glioma classification for the first time (7), and the newly released 2021 guidance (WHO CNS5) emphasized the diagnostic value of molecular diagnosis for glioma subgroup (8). This substantial change has been achieved by further advancing the role of molecular diagnosis in the classification of CNS tumors, but still relying on other established methods for diagnosis of tumor characteristics including histology and immunohistochemistry. Isocitrate dehydrogenase (IDH) is a common molecular marker in glioma and is frequently used for predicting prognosis. Prior studies have shown that the prognosis of IDH-mutant gliomas is better than IDH-wild type gliomas (9, 10). This genetic grouping serves an important clinical indicator of stratifying tumors with differential susceptibility to adjuvant treatment. The biological similarities between some LGGs and glioblastomas make it critical to identify glioblastomas and separate them from more favorable IDH-mutant entities (11).

Unfortunately, preoperative distinction between different glioma grades and subtypes remains challenging with insufficient sensitivity and specificity. In addition, in the cases that gliomas at certain specific sites cannot be resected or punctured, or in patients who cannot undergo surgery due to age or other problems, the method based on image analysis can be used as a supplementary diagnostic tool for molecular classification of gliomas, and thus may have great potential value in treatment decisions (12). Magnetic resonance imaging (MRI) has been demonstrated a promising approach to non-invasively distinguish various tumor entities (13, 14).

MRI is also the preferred imaging method for glioma. Morphological characteristics and enhancement pattern of gliomas can be obtained by conventional MRI. Prior studies have shown that gliomas with different grades and IDH mutation status have differences in lesion properties such as location, internal signal and enhancement patterns (15, 16). Several imaging biomarkers contribute to the diagnosis of molecular subtypes of gliomas. Such as, T2-FLAIR mismatch (T2FM), which is a sign demonstrated a specificity of almost 100% for IDH mutant astrocytoma in recent studies (17, 18). However, morphological indicators are difficult to be quantified, so it cannot predict glioma grades and molecular subtypes accurately. Diffusion-weighted imaging (DWI) is an important sequence of MRI and serve for the identification and differential diagnosis in a broad spectrum of cancers (19). The assessment of cancers using DWI is based on the assumption that free water motion in tissues diminishes with growing tumor cellularity (20). The calculation of apparent diffusion coefficient (ADC) maps from DWI (at least with two b values) is a fast and straightforward procedure that can support grading and have shown the capability for IDH typing in gliomas (21–23). However, it is often difficult to identify the grades or even molecular subtypes of gliomas with single indicators obtained only by conventional MRI sequences. Few studies have combined MRI morphological features and ADC values to predict glioma grades and IDH mutation status. Additionally, consideration of patient age may help diagnosis because it has been shown that IDH-wild type gliomas are more common in older patients (11).

The purpose of our study was to explore the correlation between MRI morphological characteristics, ADC parameters and glioma grades, IDH mutation status. The developed multivariate predictive models may provide a new strategy for the formulation of glioma treatment, follow-up plan and prognosis evaluation.

## Materials and Methods

### Patient Selection

A total of 166 glioma patients admitted to our hospital from 2016 to 2020 were selected, including 92 males and 74 females, aged from 14 to 85 years old, with a median age of 53 years old. There were 43 cases in LGGs (12 cases in grade I, 31 cases in grade II), 123 cases in HGGs (18 cases in grade III, 105 cases in grade IV). There were 48 IDH-mutant cases and 112 IDH-wild type cases. Inclusion criteria: (1) Meet the diagnostic criteria of glioma; (2) All patients underwent surgical treatment and obtained postoperative pathological results and molecular diagnosis results. (3) The patients underwent preoperative MRI examination with complete data. Exclusion criteria: (1) Receiving conservative treatment; (2) MRI imaging quality was poor and cannot be studied and analyzed; (3) Complicated with other neurological diseases, such as cerebral infarction, cerebral hemorrhage. All patients signed informed consent before the enhanced MRI examination according to the hospital regulations. This retrospective study was exempted from ethical review.

### MRI Parameters

MR images were acquired with a 3.0-T MRI scanner (MAGNETOM Prisma; Siemens Healthineers, Erlangen, Germany). The MRI protocols for brain tumor at our hospital included T2-weighted, T2-weighted fluid-attenuated inversion recovery (FLAIR), T1-weighted sequences before and after administration of a gadolinium-based contrast agent and DWI. The parameters of MRI scanning are attached in **Supplementary Material**. Gadolinium-diethylenetriamine pentaacetic acid (Gd-DTPA) as injected *via* the cubital vein mass with a high-pressure syringe at a dose of 0.1 mmol per kilogram of body weight and a flow rate of 5 mL/s. Enhanced T1W scanning was performed at axial, coronal, and sagittal positions.

The ADC map is created by dividing the signal from the trace-DWI image by the signal from each corresponding point in the b0 image and taking logarithms:


$$ADC=–\frac{1}{b_{1}}\times In(S_{b1}/S_{b0})$$

Where ADC stands for apparent diffusion coefficient, b0 = 0 s/mm^2^ and b1 = 1000 s/mm^2^, *S_b0_* and *S_b1_* are the signal intensities of each image at b0 and b1. ADC maps are mathematically calculated using the inline technique, as the pure display of consolidated ADC values.

To ensure high-quality ADC maps, the DWI sequence was optimized to maximize signal noise ratio (SNR) and reduce artifacts that may be caused by motion, B0 inhomogeneity, chemical shifts, Nyquist ghosting, susceptibility effects, and noise amplification. Eddy current of the diffusion-encoding gradient was minimized by using a twice-refocused bipolar diffusion preparation. In order to get the ideal ADC map, the noise level was set to 40, as recommended by the equipment manufacturer. Correspondingly, we can get the ADC values from the ADC map by drawing regions of interest (ROIs).

### Image Analysis

Two radiologists with more than 10 years of experience in radiology independently reviewed the MR images. The disagreements were resolved through consultation. The morphological signs of MRI were observed, including (1) hemorrhage; (2) cystic lesion; (3) tumor boundary, including clear or blur; (4) peritumoral edema, including no edema, mild edema (the longest diameter of edema < the longest diameter of the tumor), severe edema (the longest diameter of edema ≥ the longest diameter of the tumor); (5) enhancement pattern, including no enhancement, patchy enhancement and rim enhancement; and (6) distribution of lesions, which were divided into the single lobe, trans-lobe growth with corpus callosum involvement, trans-lobe growth with insula involvement, trans-lobe growth (neither corpus callosum involvement nor insula involvement), thalamus or brain stem.

**Figure 1:**
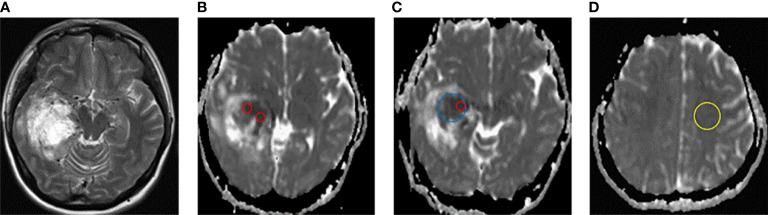
Schematic diagram of ADC measurement. **(A)** T2W axial map of IDH-wild type glioblastoma of the right temporal lobe. **(B–D)** ADC diagram ROI delineates sketch. ADC_min_ (ROI of 3 lowest visual ADC values for each patient, red circle), ADC_mean_ (maximum cross-section of axial solid tumor, blue circle), and ADC_nawm_ (contralateral hemisphere centrum semiovale normal appearing white matter, yellow circle).

In this study, ADC values and derived parameters were measured at Syngo. *Via* workstation (Siemens healthineers, Erlangen, Germany), including (1) Minimum apparent diffusion coefficient (ADC_min_): Three different 20-30 mm^2^ ROIs) of visually lowest ADC values were outlined in each tumor, and the average value was ADC_min_. (2) Mean apparent diffusion coefficient (ADC_mean_): The ROI is plotted as large as possible on the largest layer of tumor transverse axis entity components, avoiding necrosis, cystic degeneration, calcification, vessels, etc., and the ADC value is measured as ADC_mean_. (3) Map ROI in the contralateral hemispherical center of the normal white matter and measure ADC (ADC_nawm_). ADC_min_/ADC_nawm_ was denoted as rADC_min_ (relative ADC_min_). (4) ADC_mean_/ADC_nawm_ was denoted as rADC_mean_ (relative ADC_mean_). The average values of the above parameters measured by 2 physicians were obtained. ADC ROI is outlined in **Figure 1**.

### Histopathologic Analysis

All tissue specimens were fixed into paraffin blocks and analyzed in the Pathology Department of our hospital. The tumors were classified into grade I, II, III and IV, according to 2016 WHO Classification of Tumors of the Central Nervous System (glioma-related classification and grading). The IDH mutation status of tumor specimens were detected by immunohistochemical examination and determined according to the combination of the specimen with the monoclonal antibody that can detect IDH1 gene R132H point mutation in glioma. Positive IDH1 expression was defined as IDH-mutant group, and negative IDH1 expression was defined as IDH-wild type group.

### Statistical Analysis

The statistical analysis was conducted using SPSS 22.0. The Shapiro-Wilk test was carried out to test the normality of continuous variables. Since all continuous variables in this study were normally distributed, they were described as mean ± standard deviations (SDs) and compared by Student’s t-test. The categorical variables were described by number and percentage (%) and compared by Chi-square tests. For the dependent variables of dichotomies or disordered multiclassification, Pearsonχ^2^ test or exact probability method was used to compare the differences between the two groups, including the Holm-Bonferroni correction of multiple tests. Wilcoxon rank-sum test was used to compare the difference between the two groups for the ordered multi-classification dependent variable (degree of peritumoral edema). The receiver-operating characteristic curve (ROC) analysis was performed to evaluate the diagnostic performance of the developed models. Variables with *P* < 0.05 in the univariate analysis were included in multivariate logistic regression analysis and model construction. *P* < 0.05 indicated that the difference was statistically significant.

## Results

### Patient Demographics

A total of 166 patients were included in the analysis. There was no gender difference between low-grade and high-grade glioma patients (*P* = 0.172). The age of high-grade glioma patients (55.5 ± 14.5) was higher than that of low-grade glioma group (38.6 ± 12.8) (*P* < 0.05). IDH mutation was more common in LGGs than that in the HGGs (48.8% vs. 23.5%) (*P* < 0.05) (**Table 1**).

**Table 1:** Demographics of the study population.

Variables		Total	LGGs (n = 43)	HGGs (n = 123)	*t*/*χ^2^* value	*P* value
Gender	Male	92	20 (46.5%)	72 (58.5%)	1.865	0.172
	Female	74	23 (53.5%)	51 (41.5%)		
Age (years)		51.1 ± 15.9	38.6 ± 12.8	55.5 ± 14.5	-6.783	< 0.001^***^
IDH mutant status^#^	IDH^mut^	48	20 (48.8%)	28 (23.5%)	9.259	0.002^**^
	IDH^wt^	112	21 (51.2%)	91 (76.5%)		

^#^Six patients had no IDH status available for assessment. LGGs, low-grade gliomas; HGGs, high-grade gliomas.

Significance level markers P < 0.01^**^, P < 0.001^***^.

### Comparison of MRI Morphological Characteristics Between LGGs Group and HGGs Group

Compared with LGGs, HGGs were more prone to have hemorrhage (*P* < 0.01) and cystic lesion (*P* < 0.05) (**Table 2**). In addition, the peritumoral edema was more severe (*P* < 0.001), and tumor boundaries were less clear (*P* < 0.01) in HGGs than those in LGGs. In terms of enhancement pattern, HGGs were more likely to show rim enhancement, while LGGs were more likely to show no obvious enhancement (*P* < 0.001). Regarding the distribution of lesions, a single lobe (41.9%) was more frequently to be observed in LGGs, while HGGs were more likely to show cross-lobe growth (66.6%) with corpus callosum and insula. There were significant differences in the distribution and location of lesions between two different grades of gliomas (*P* < 0.001) (**Table 2**).

**Table 2:** Comparison of MRI morphological characteristics of LGGs and HGGs.

Parameters		LGGs (*n* = 43)	HGGs (*n* = 123)	*χ^2^*/*Z* value	*P* value
Hemorrhage^#^	Present	1 (2.3%)	29 (24.4%)	9.900	0.002^**^
	None	41 (97.7%)	90 (75.6%)		
Cystic lesion^†^	Present	15 (34.9%)	64 (54.2%)	4.723	0.030^*^
	None	28 (65.1%)	54 (45.8%)		
Peritumoral edema	None	25 (58.1%)	18 (14.6%)	-5.010	< 0.001^***^
	Mild	8 (18.6%)	35 (28.5%)		
	Severe	10 (23.3%)	70 (56.9%)		
Tumor boundary	Clear	28 (65.1%)	49 (39.8%)	8.187	0.004^**^
	Blur	15 (34.9%)	74 (60.2%)		
Enhancement pattern^‡^	No enhancement	27 (62.8%)	13 (10.7%)	52.773	< 0.001^***^
	Patchy enhancement	13 (30.2%)	41 (33.9%)		
	Rim enhancement	3 (7.0%)	67 (55.4%)		
Distribution of lesions	Single lobe	18 (41.9%)	28 (22.8%)	20.940	< 0.001^***^
	Trans-lobe growth with corpus callosum involvement	5 (11.6%)	26 (21.1%)		
	Trans-lobe growth with insula involvement	5 (11.6%)	34 (27.6%)		
	Trans-lobe growth	2 (4.7%)	22 (17.9%)		
	Thalamus or brain stem	13 (30.2%)	13 (10.6%)		

^#^Hemorrhage status was evaluated as uncertain in a total of 5 patients.

^†^Cystic lesion status was evaluated as uncertain in a total of 5 patients.

^‡^Two patients did not undergo MRI enhancement examination.

Significance level markers P < 0.05*, P < 0.01**, P < 0.001***.

### Comparison of Morphological Characteristics Between IDH-Mutant Group and IDH-Wild Type Group

**Figure 2:**
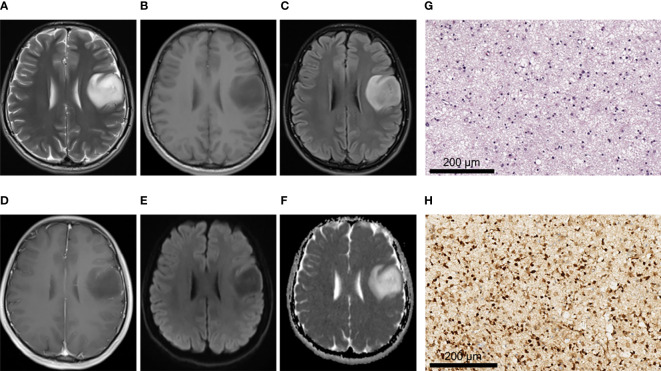
A 28-year-old male patient with left frontal oligodendroglioma, WHO grade II, IDH-mutant type. **(A–C)** MRI axial T2W, T1W, and T2-FLAIR sequences showed clear tumor boundary, no cystic lesion, no hemorrhage, and no obvious edema around the tumor. **(D)** T1 postcontrast showed no obvious enhancement. **(E, F)** When b value was 1000, tumor was unrestricted diffusion in DWI and ADC images. **(G)** HE staining showed moderate increase in cell density, a small amount of nuclear atypia and loose background (×200). **(H)** IDH1 positive expression (×200).

**Figure 3:**
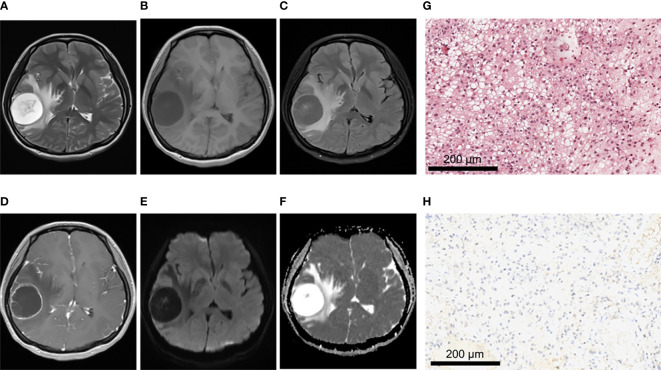
A 21-year-old female patient with right frontoparietal temporal lobe pilocytic astrocytoma, WHO grade I, IDH-wild type. **(A–C)** MRI axial T2W, T1W, T2-FLAIR sequences showed clear tumor boundary, no hemorrhage, and severe edema around the tumor. **(D)** T1 postcontrast showed thin wall ring enhancement. **(E, F)** When b value was 1000, tumor was unrestricted diffusion in DWI and ADC images. **(G)** HE staining showed moderate cell density with oligodendrocyte like changes and a focal myxoid background (×200). **(H)** IDH1 negative expression (×200).

**Figure 4:**
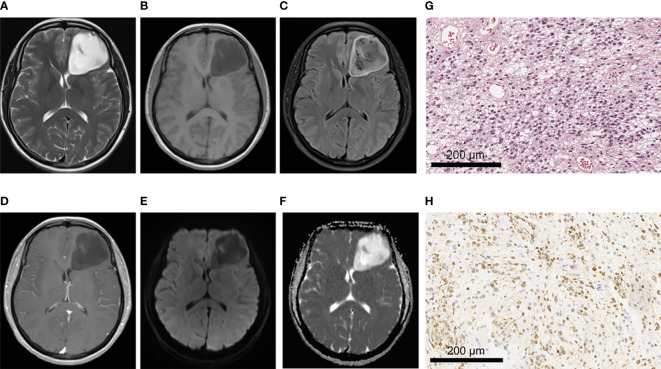
A 43-year-old female patient with left frontal anaplastic astrocytoma, WHO grade III, IDH-mutant type. **(A–C)**MRI axial T2W, T1W, T2-FLAIR sequences showed clear tumor boundary, slightly uneven internal signal and no peritumor edema. **(D)** T1 postcontrast showed no obvious enhancement. **(E, F)** When b value is 1000, tumor was locally and slightly restricted diffusion in DWI and ADC images. **(G)** HE staining showed moderate to severe increase in cell density, accompanied by nuclear atypia and mitotic images (×200). **(H)** IDH1 positive expression (×200).

**Figure 5:**
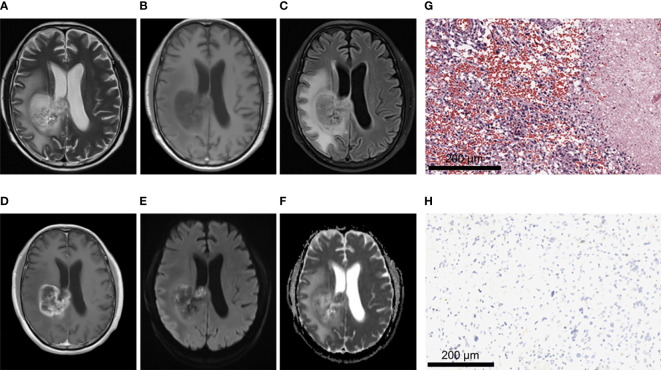
A 62-year-old male patient with glioblastoma of the right fronto-parietal lobe with corpus callosum involvement, WHO grade IV, IDH-wild type. **(A–C)** MRI axial T2W, T1W, T2-FLAIR sequences showed multiple cystic lesions within the tumor, with uneven signals and severe peritumor edema. **(D)** T1 postcontrast showed obvious irregular and thick rim enhancement. **(E, F)** When b value is 1000, tumor was locally obvious restricted diffusion in DWI and ADC images. **(G)** HE staining showed increased cell density and marked atypia, accompanied by extensive necrosis (×200). **(H)** IDH1 negative expression (×200).

There were no significant differences between IDH-mutant gliomas and IDH-wild type gliomas in hemorrhage, cystic lesion, peritumoral edema and tumor boundary (All *P* > 0.05) (**Table 3**). In terms of enhancement pattern, IDH-wild type gliomas were more likely to be characterized by rim enhancement, while IDH mutant gliomas were more likely to be characterized by no obvious enhancement (*P* < 0.001). There were significant differences in the distribution of lesions between gliomas patients with and without IDH mutation (*P* < 0.01). Compared with IDH-wild type gliomas, IDH-mutant gliomas were more likely to be associated with insula involvement (*P* < 0.001). IDH-mutant cases tended to have one lobe, and the lesions were mostly located in the frontal lobe (11/15, 73.3%) (**Table 3**). The representative MRI features and pathological characteristics of gliomas of different IDH molecular subtypes at different grades are shown in **Figures 2**–**5**.

**Table 3:** Comparison of MRI morphological characteristics between IDH-mutant and IDH-wild type gliomas.

Parameters		IDH^mut^ (*n *= 48)	IDH^wt^ (*n* = 112)	*χ^2^*/*Z* value	*P* value
Hemorrhage	Present	8 (16.7%)	22 (20.6%)	0.322	0.570
	None	40 (83.3%)	85 (79.4%)		
Cystic lesion	Present	19 (39.6%)	56 (52.3%)	2.158	0.142
	None	29 (60.4%)	51 (47.7%)		
Peritumoral edema	None	12 (25.0%)	29 (25.9%)	-7.767	0.443
	Mild	16 (33.3%)	25 (22.3%)		
	Severe	20 (41.7%)	58 (51.8%)		
Tumor boundary	Clear	21 (43.8%)	50 (56.3%)	0.011	0.917
	Blur	27 (44.6%)	62 (55.4%)		
Enhancement pattern	No enhancement	23 (48.9%)	16 (14.4%)	26.943	< 0.001^***^
	Patchy enhancement	16 (34.0%)	34 (30.6%)		
	Rim enhancement	8 (17.0%)	61 (55.0%)		
Distribution of lesions	Single lobe	15 (31.3%)	30 (26.8%)	14.915	0.005^**^
	Trans-lobe growth with corpus callosum involvement	9 (18.8%)	21 (18.8%)		
	Trans-lobe growth with insula involvement	19 (39.6%)	22 (17.9%)		
	Trans-lobe growth	3 (6.3%)	19 (17.0%)		
	Thalamus or brain stem	2 (4.2%)	22 (19.6)		

Significance level markers P < 0.01**, P < 0.001***.

### Comparison of ADC Values and Derived Parameters Between LGGs Group and HGGs Group

**Figure 6:**
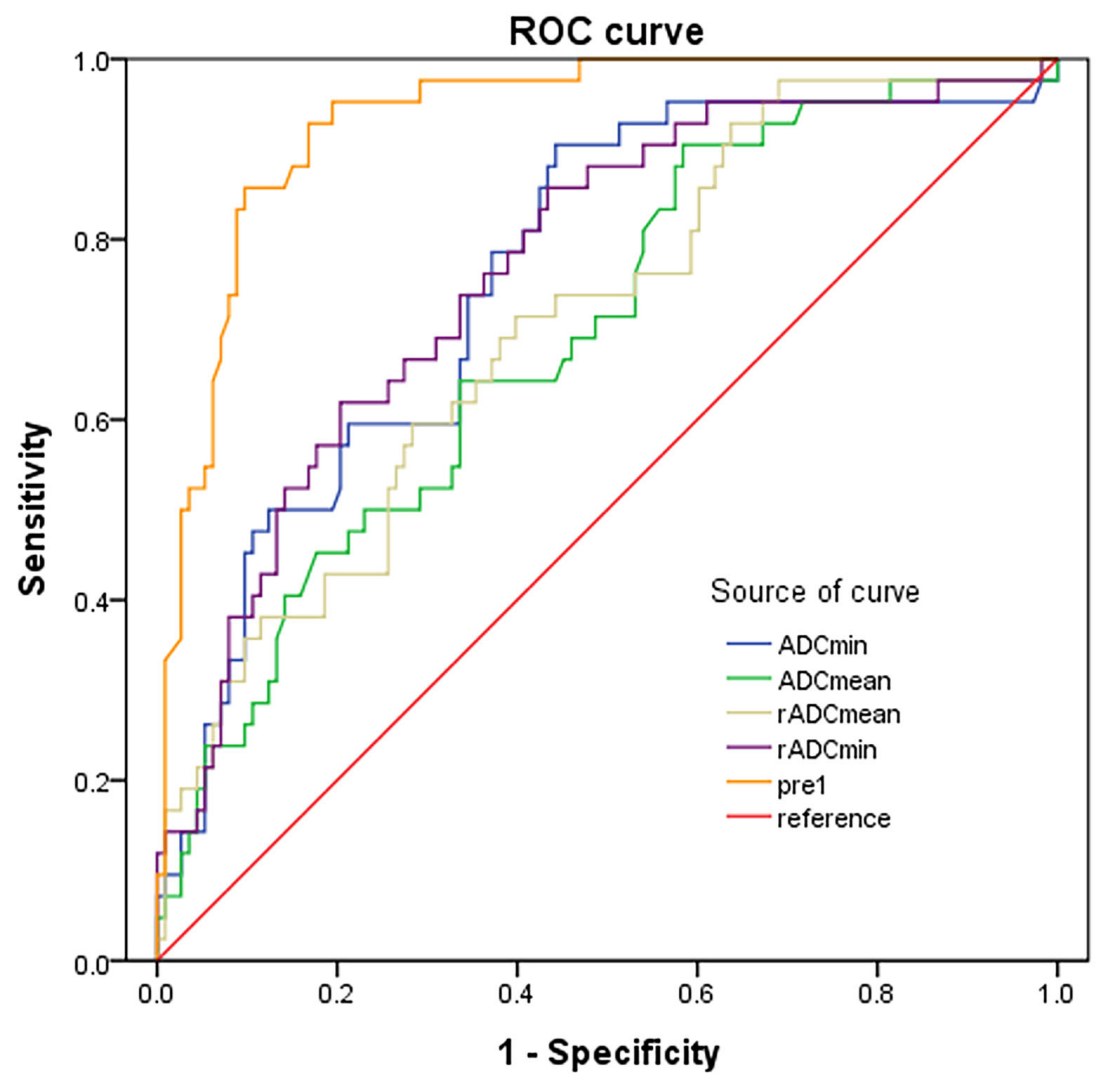
ROC curves of ADC indicators and combined predictors for differentiating LGGs from HGGs.

ADC_min_, ADC_mean_, rADC_min_ and rADC_mean_ of HGGs were significantly lower than those of LGGs (All *P* < 0.001) (**Table 4**). ROC curve analysis was then performed to differentiate LGGs from HGGs using ADC indicators (**Figure 6**). It was found that the diagnostic efficiency of rADCmin was higher than that of ADCmin, and rADCmean was higher than that of ADCmean. Among four different ADC parameters measured, rADC_min_ had the highest diagnostic efficiency in differentiating LGGs from HGGs, with an AUROC of 0.775 (95% CI=0.695-0.856), the diagnostic optimal cut-off value of 1.26×10^-3^ mm^2^/s, the sensitivity of 62.79%, specificity of 80.49%, and Yuden index of 0.443 (**Table 4**).

**Table 4:** Comparison and ROC curve analysis of ADC parameters between LGGs and HGGs.

ADC parameters (×10^-3^ mm^2^/s)	LGGs (*n* = 43)	HGGs (*n* = 123)	*t* value	*P* value	AUC (95% *CI*)	Cut-off value	Sensitivity (%)	Specificity (%)	Yuden index
ADC_min_	1.03 ± 0.30	0.80 ± 0.21	4.626	< 0.001***	0.767 (0.686-0.849)	0.79	90.70	56.91	0.476
ADC_mean_	1.23 ± 0.31	1.04 ± 0.25	4.130	< 0.001***	0.697 (0.608-0.786)	0.96	90.70	57.72	0.330
rADC_min_	1.39 ± 0.40	1.05 ± 0.30	5.816	< 0.001***	0.775 (0.695-0.856)	1.26	62.79	80.49	0.443
rADC_mean_	1.66 ± 0.44	1.35 ± 0.36	4.571	< 0.001***	0.710 (0.622-0.797)	1.41	72.09	60.98	0.331

AUC, Area under the curve; CI, Confidence Interval.

Significance level markers P < 0.001***.

### Comparison of ADC Values and Derivative Parameters Between IDH-Mutant Group and IDH-Wild Type Group

**Figure 7:**
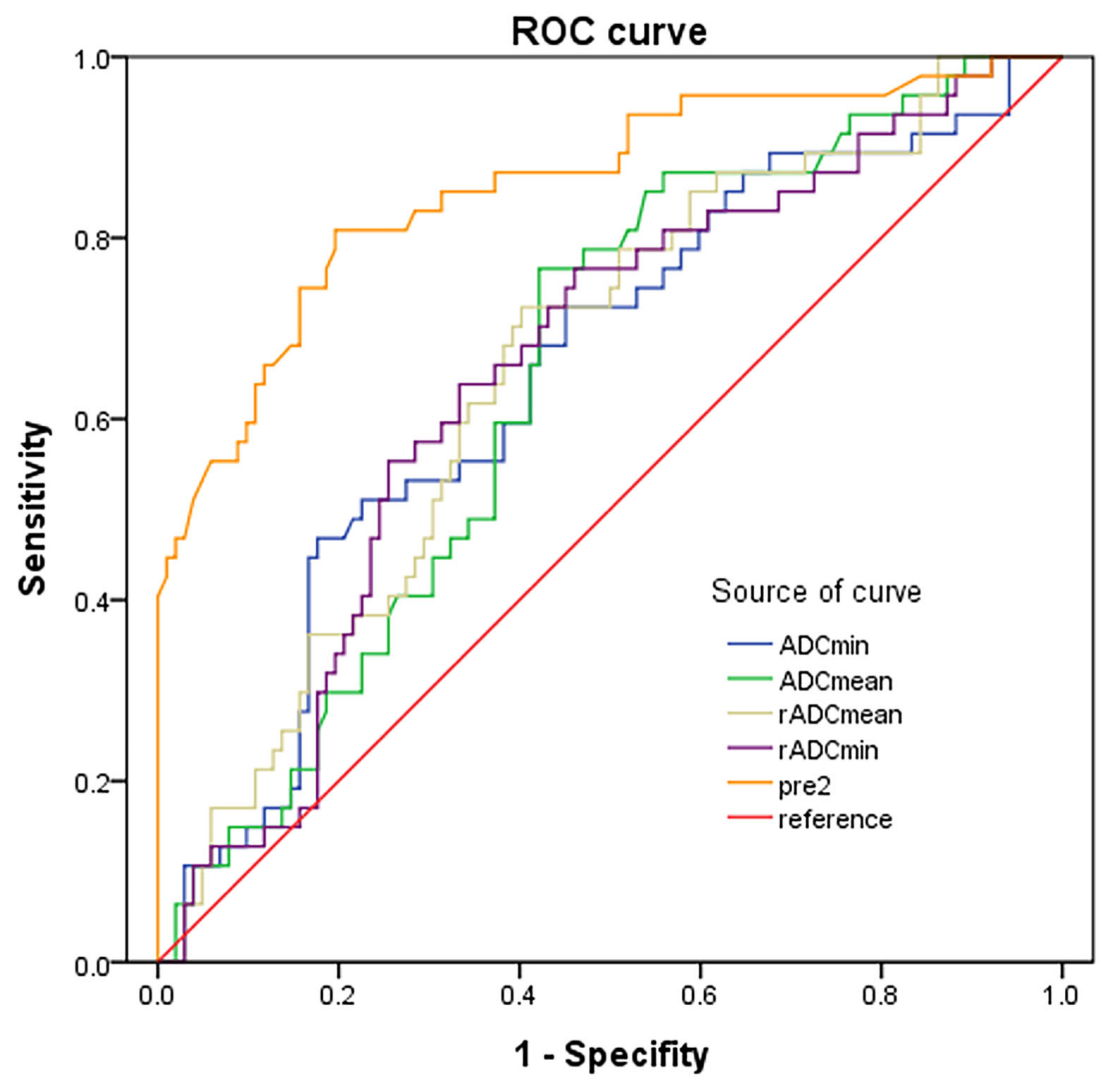
ROC curves of ADC indicators and combined predictors for distinguishing IDH mutants from IDH-wild type gliomas.

The ADC_min_, ADC_mean_, rADC_min_ and rADC_mean_ of IDH-wild type gliomas were significantly lower than those of IDH-mutant gliomas, and the differences were statistically significant (*P* < 0.05) (**Table 5**). The diagnostic performance of ADC parameters for distinguishing gliomas patients with and without IDH mutation was then evaluated by the ROC analysis (**Figure 7**). Analysis showed that the diagnostic efficiency of rADCmin was higher than that of ADCmin, and rADCmean was higher than that of ADCmean. Similar to the differentiation of glioma grades, rADC_min_ had the highest diagnostic efficiency in differentiating IDH-mutant gliomas from IDH-wild type gliomas, with the diagnostic optimal cut-off value of 1.14×10^-3^ mm^2^/s, an AUROCC of 0.656 (95% CI=0.566-0.746), the sensitivity of 62.5%, specificity of 66.96%, and Yoden index of 0.295 (**Table 5**).

**Table 5:** Comparison and ROC curve analysis of ADC parameters between IDH^mut^ and IDH^wt^ gliomas.

ADC parameters (×10^-3^ mm^2^/s)	IDH^mut^ (*n* = 48)	IDH^wt^ (*n* = 112)	*t* value	*P* value	AUC (95%*CI*)	Cut-off value	Sensitivity (%)	Specificity (%)	Yuden index
ADC_min_	0.94 ± 0.24	0.83 ± 0.26	2.429	0.016^*^	0.653 (0.561-0.745)	0.98	45.83	83.04	0.289
ADC_mean_	1.18 ± 0.25	1.05 ± 0.29	2.611	0.010^*^	0.643 (0.555-0.731)	1.05	75.00	58.04	0.330
rADC_min_	1.59 ± 0.37	1.37 ± 0.42	2.941	0.004^**^	0.656 (0.566-0.746)	1.14	62.50	66.96	0.295
rADC_mean_	1.25 ± 0.33	1.09 ± 0.37	2.634	0.009^**^	0.652 (0.562-0.742)	1.40	70.83	59.82	0.307

Significance level markers P < 0.05*, P < 0.01**.

### Multivariate Logistic Regression Analysis

We next tested the multivariate models by combining all the above significant factors. Age was divided into two groups: < 60 years old and ≥ 60 years old. Relative ADC parameters (rADC_mean_ and rADC_min_) were divided into two groups according to the optimal cut-off value in single factor analysis. The Noninvasive Grading Model for predicting glioma grades included age, rADC_mean_, rADC_min_, cystic lesion, hemorrhage, tumor boundary, peritumoral edema, lesion distribution and enhancement pattern. And the predictor factor 1 (pre1) were generated. The Noninvasive IDH Genotyping Model for predicting glioma IDH mutation status generated pre2, containing age, rADC_mean_, and rADC_min_, lesion distribution and enhancement pattern.

We found that age (≥ 60 years), rADC_min_ (< 1.26×10^-3^ mm^2^/s), rim enhancement, and lesion distribution (thalamus or brainstem) were independent risk factors for predicting HGGs (**Table 6**). Age (≥ 60 years), rim enhancement, and lesion distribution (trans-lobe growth with corpus callosum involvement) were independent risk factors for IDH-wild type gliomas (**Table 7**). The accuracy of the multivariate logistic regression model combining age, morphological characteristics and ADC parameters in predicting glioma grades and IDH mutation status was improved compared with that of a single indicator (**Figures 6**, **7**). The Noninvasive Grading Model showed an AUROC of 0.934 (95% CI=0.895-0.973), a sensitivity of 91.2%, and a specificity of 78.6% in differentiating HGGs from LGGs. The AUROC of the Noninvasive IDH Genotyping Model was 0.857 (95% CI=0.787-0.926), with a sensitivity of 88.2% and specificity of 63.8% (**Table 8**).

**Table 6:** Multivariate logistic regression analysis of glioma grades.

Variables	Noninvasive Grading Model		
	*OR*	95%*CI*	*P* value
**Age (≥ 60 years)**	7.877	1.359~45.638	0.021^*^
**rADCmean (< 1.41)**	1.256	0.290~5.446	0.761
**rADCmin (< 1.26)**	4.548	1.162~17.799	0.030^*^
**Lesion distribution**			
Single lobe	Reference		
Trans-lobe growth with corpus callosum involvement	0.831	0.118~5.860	0.853
Trans-lobe growth with insula involvement	3.352	0.652~17.222	0.147
Trans-lobe growth	2.795	0.284~27.535	0.379
Thalamus or brain stem	0.144	0.024~0.868	0.034^*^
**Enhancement pattern**			
No enhancement	Reference		
Patchy enhancement	5.523	1.410~21.629	0.140
Rim enhancement	41.594	5.810~297.794	< 0.001^***^
**Cystic lesion**	2.867	0.758~10.838	0.121
**Hemorrhage**	6.426	0.465~88.896	0.165
**Tumor boundary**	1.390	0.408~4.744	0.599
**Peritumoral edema**			
None	Reference		
Mild	2.376	0.529~10.544	0.259
Severe	0.501	0.085~2.957	0.446

OR, odds ratio.

Significance level markers P < 0.05*, P < 0.001***.

**Table 7:** Multivariate logistic regression analysis of IDH status of glioma.

Variables	Noninvasive IDH Genotyping Model		
	*OR*	95%*CI*	*P* value
**Age (≥ 60years)**	3.690	1.235~11.029	0.019^*^
**rADCmean (< 1.40)**	1.868	0.594~5.875	0.285
**rADCmin (< 1.14)**	1.593	0.488~5.197	0.441
**Lesion distribution**			
Single lobe	Reference		
Trans-lobe growth with corpus callosum involvement	0.266	0.085~0.835	0.023^*^
Trans-lobe growth with insula involvement	0.389	0.106~1.432	0.156
Trans-lobe growth	1.131	0.226~5.669	0.881
Thalamus or brain stem	4.673	0.856~25.497	0.075
**Enhancement pattern**			
No enhancement	Reference		
Patchy enhancement	2.348	0.844~6.535	0.102
Rim enhancement	6.371	1.931~21.016	0.002^**^

Significance level markers P < 0.05*, P < 0.01**.

**Table 8:** Diagnostic efficiency of multivariate Logistic regression model.

Model	AUC (95%*CI*)	Sensitivity (%)	Specificity (%)	PPV (%)	NPV (%)	Yuden index
Noninvasive Grading Model	0.934 (0.895-0.973)	91.2	78.6	92.0	76.7	0.698
Noninvasive IDH Genotyping Model	0.857 (0.787-0.926)	88.2	63.8	84.1	71.4	0.520

PPV, Positive Predictive Value; NPV, negative predictive value.

## Discussion

The malignancy of glioma determines the choice of the surgical treatment plan and the prognosis of patients. The higher the tumor grade is, the worse the prognosis is. However, a single histopathological grade often has limitations. As in WHO grade IV glioblastoma, the degree of malignancy and prognosis may be different with different IDH gene types. WHO CNS5 in 2021 introduces a series of molecular diagnostic indicators on the basis of histological diagnosis, forming an integrated diagnosis and hierarchical reporting system, and defining multiple tumor types and subtypes (8). IDH gene family is still an important molecular marker of adult diffuse glioma. But something has changed. Previously, glioblastoma was diagnosed based on histological findings of microvascular proliferation and/or necrosis, including IDH mutations (10%) and IDH wild-type tumors (90%). In WHO CNS5, glioblastoma will contain only IDH wild-type tumors. IDH is a key rate-limiting enzyme of the tricarboxylic acid cycle (TCA), and IDH gene mutation in most gliomas occurs at the R132H site of IDH1 (24). Studies have shown that IDH mutation is an early event of glioma formation and has an important impact on glioma progression and tumor behavior (25). The clinical outcome of the IDH-mutant group is often better than that of the IDH-wild type group (26), and the IDH-wild type group is more aggressive, similar to the biological behavior of glioblastoma (17). Histopathological and immunohistochemical analysis is the final criteria for grade diagnosis and molecular subtype diagnosis of glioma, but there are the following limitations: (1) Internal heterogeneity and sampling bias of glioma may lead to errors in pathological results (27, 28). (2) The delayed diagnosis is not conducive to the formulation of surgical plan and the selection of preoperative treatment plan. (3) Some patients obtain pathological results by biopsy before surgery, but this is an invasive procedure, which may induce cerebral hemorrhage, epilepsy and other complications and increase the risk of iatrogenic injury (29, 30). MRI examination is an important auxiliary diagnostic method for glioma. MRI manifestations of glioma with different grades and IDH mutation status also have their characteristics. MRI can provide rich information for the diagnosis and prognosis evaluation of glioma. In this study, we successfully developed two noninvasive models by combing multiple new MRI features to distinguish low- and high-grade gliomas as well as with and without IDH mutation. These multivariate models led to a better predictive performance for glioma severity and IDH mutation than the single predictor.

In addition to IDH, many other molecular markers of glioma have been studied more and more in recent years, such as 1p/19q co-deletion, MGMT promoter methylation, TP53 mutation, EGFR amplification, etc. (31). These molecular markers have been confirmed to be related to the prognosis and treatment response in glioma patients. And in the fifth edition of the guidelines (WHO CNS5), glioblastoma, IDH-wildtype should be diagnosed in the setting of an IDH-wildtype diffuse and astrocytic glioma in adults if there is microvascular proliferation or necrosis or TERT promoter mutation or EGFR gene amplification or +7/-10 chromosome copy (8). Therefore, the importance of molecular markers for the diagnosis of glioma has once again attracted strong attention. However, it is difficult to predict the molecular subtypes of glioma with high accuracy by conventional MRI technology and general image post-processing simply. In recent years, classical machine learning approaches and deep learning approaches have shown the ability to identify the predictive features and to perform the actual prediction (32). Deep learning technology achieving performance that exceeds humans in the identification of content in images. So it can see the unseeable to predict molecular markers from MRI of brain gliomas. It is believed that a more reliable model can be used to better identify molecular markers of glioma through the combination of MRI and machine learning in the future.

Aging is usually associated with a poor prognosis of glioma. Consistent with the recent study, in our study, we found that high-grade glioma patients were older than patients with low-grade glioma (33). We further showed that age ≥ 60 years was an independent risk factor for predicting HGGs and IDH-wild type gliomas. A previous study reported that IDH-wild type gliomas are more common in elderly patients (34). We also found that the age of IDH-wild type gliomas was higher than that of IDH-mutant gliomas.

MRI morphological characteristics of gliomas with different grades and IDH mutation status may differ greatly. In this study, HGGs are more prone to cystic lesions and hemorrhage, which may be related to the high microvascular density and strong invasiveness of HGGs. We did not observe significant difference between IDH-mutant gliomas and IDH-wild type gliomas in terms of the cystic lesion, hemorrhage and peritumoral edema. However, Lasocki et al. showed that the proportion of edema was statistically significant between IDH-mutant gliomas and IDH-wild type gliomas. All five IDH-mutant patients had an edema rate of 5-33%, whereas most IDH-wild type patients had an edema rate of > 33% (44% of IDH-wild type patients had an edema rate of 34-67% and 14% had an edema rate of 68-95%) (35). The differences may be related to the subjective classification of the degree of edema and the selection bias of the enrolled cases.

The enhancement of glioma mainly depends on the degree of damage to the blood-brain barrier. The contrast agent retention in abnormal angiogenesis thus produces characteristic enhanced images (36). Therefore, MRI enhancement features are of high value for the judgment of the malignant degree of gliomas. In this study, most HGGs and IDH-wild type gliomas showed rim enhancement, while most LGGs and IDH-mutant gliomas showed hypovascular, which is basically consistent with previous literature reports (3, 11). Lasocki et al. suggested that unenhanced tumor volume > 33% was associated with IDH-mutant glioblastoma (35). Tumor location and distribution of lesions are one of the important factors affecting the prognosis of glioma patients. In this study, it was found that LGGs are more common to involve a single lobe, while HGGs are more common to involve multiple lobes, and more likely to involve corpus callosum and insula. The differences in the distribution of lesions in gliomas with different IDH mutation status have also been reported in previous literature. Nakae et al. showed that tumor location in the unilateral frontal lobe was highly correlated with IDH-mutant gliomas (P < 0.001) (37). Goze et al. found that 100% of LGGs with insula centers were IDH mutants (38). In our study, we also showed that compared with IDH-wild type gliomas, IDH-mutant gliomas were more likely to be associated with insula involvement, and frontal lobe involvement was more common in cases involving the single lobe.

A meta-analysis showed that ADC was significantly negatively correlated with the number of tumor cells in gliomas (39). The results of our study are consistent with previous studies, and the four ADC parameters of HGGs are lower than those of LGGs. The white matter cell substructure of HGGs was greatly damaged, and the diffusion of water molecules was limited. Therefore, In DWI imaging, HGGs showed higher signals, and LGGs showed lower signals. And accordingly, HGGs had lower ADC values (40, 41). In addition, The ADC value of IDH-wild type gliomas was significantly lower than that of IDH-mutant gliomas (13, 22, 23), which was consistent with the results obtained in this study. Patel et al. noted that histologically observed microcysts tended to increase in IDH mutant LGG cases, which could explain the higher ADC values in these cases, but the pathophysiological mechanism needed to be further elucidated (17).

In this study, we demonstrated the effectiveness of the combination of ADC parameters and MRI morphological features in predicting glioma grades and IDH molecular subtypes. The two multivariate logistic regression models combined with age, ADC parameters and MRI morphological qualitative parameters performed better in distinguishing the LGGs group from the HGGs group and the IDH-mutant group from the IDH-wild type group than using any single ADC value parameter alone.

However, this study also had some limitations, such as (1): This study was a single-center retrospective analysis, and the number of enrolled cases was relatively small. (2) Conventional MRI sequences and enhanced sequence were used instead of multimodality MRI imaging to obtain multi-parameter characteristics of tumors. Compared with other diffusion imaging, ADC has some limitations. (3) Subjective judgment errors may occur in the judgment of certain imaging features. For example, in some cases, it is difficult to distinguish simple vasogenic edema from non-enhanced tumors.

In conclusion, gliomas with different grades and IDH mutation status had significant differences in MRI morphology and ADC parameters. By combining age, MRI morphological characteristics and ADC value parameters, the accuracy of predicting histopathological grades and IDH molecular subtypes of glioma was greatly improved.

## Data Availability Statement

The raw data supporting the conclusions of this article will be made available by the authors, without undue reservation.

## Ethics Statement

All patients signed written informed consent before the enhanced MRI examination according to the regulations of Huadong Hospital, Fudan University. This retrospective study was exempted from ethical review and were conducted in accordance with the World Medical Association Declaration of Helsinki-Ethical Principles for Medical Research Involving Human Subjects.

## Author Contributions

SL and XF conceived and presented idea. ND, XZ and YY collected the data. RM, WS, LX, YS and GL analyzed the data. XX provided statistical guidance. ND drafted the manuscript. All authors reviewed the manuscript, and SL made corrections to the manuscript. All authors contributed to the article and approved the submitted version.

## Conflict of Interest

The authors declare that the research was conducted in the absence of any commercial or financial relationships that could be construed as a potential conflict of interest.

## Publisher’s Note

All claims expressed in this article are solely those of the authors and do not necessarily represent those of their affiliated organizations, or those of the publisher, the editors and the reviewers. Any product that may be evaluated in this article, or claim that may be made by its manufacturer, is not guaranteed or endorsed by the publisher.
